# Fat embolism: the hidden murder for trauma patients!

**DOI:** 10.1590/0100-6991e-20243690-en

**Published:** 2024-04-17

**Authors:** MALAK BENTALEB, MOHAMMED ABDULRAHMAN, MARCELO AUGUSTO FONTENELLE RIBEIRO-JUNIOR

**Affiliations:** 1 - College of Medicine and Health Sciences, Khalifa University, Department of Surgery - Abu Dhabi - Abu Dhabi - Emirados Árabes Unidos; 2 - Sheikh Shakhbout Medical City, Division of Trauma, Critical Care and Acute Care Surgery - Department of Surgery - Abu Dhabi - Abu Dhabi - Emirados Árabes Unidos; 3 - PUC-Sorocaba, Disciplina de Cirurgia Geral e do Trauma - Sorocaba - SP - Brasil

**Keywords:** Embolism, Fat, Respiratory Distress Syndrome, Postoperative Complications, Multiple Trauma, Wounds and Injuries, Embolia Gordurosa, Insuficiência Respiratória, Complicações Pós-Operatórias, Traumatismo Múltiplo, Ferimentos e Lesões

## Abstract

**Introduction:**

fat embolism syndrome (FES) is an acute respiratory disorder that occurs when an inflammatory response causes the embolization of fat and marrow particles into the bloodstream. The exact incidence of FES is not well defined due to the difficulty of diagnosis. FES is mostly associated with isolated long bone trauma, and it is usually misdiagnosed in other trauma cases. The scope of this study was to identify and search the current literature for cases of FES in nonorthopedic trauma patients with the aim of defining the etiology, incidence, and main clinical manifestations.

**Methods:**

we perform a literature search via the PubMed journal to find, summarize, and incorporate reports of fat embolisms in patients presenting with non-orthopedic trauma.

**Results:**

the final literature search yielded 23 papers of patients presenting with fat embolism/FES due to non-orthopedic trauma. The presentation and etiology of these fat embolisms is varied and complex, differing from patient to patient. In this review, we highlight the importance of maintaining a clinical suspicion of FES within the trauma and critical care community.

**Conclusion:**

to help trauma surgeons and clinicians identify FES cases in trauma patients who do not present with long bone fracture, we also present the main clinical signs of FES as well as the possible treatment and prevention options.

## RESUMO

**Introdução:**

a síndrome da embolia gordurosa (SEG) representa um distúrbio respiratório agudo que ocorre quando uma resposta inflamatória leva a uma embolização de partículas de gordura e medula na corrente sanguínea. A incidência exata da SEG não está bem estabelecida devido à dificuldade de diagnóstico. Tal síndrome está associada principalmente a traumas isolados de ossos longos e geralmente é diagnosticada erroneamente em outros casos de trauma. O escopo deste estudo foi de realizar uma pesquisa e identificar na literatura atual casos de SEG em pacientes com trauma de natureza não ortopédica com o objetivo de definir a etiologia, a incidência e as principais manifestações clínicas.

**Métodos:**

foi realizada uma pesquisa na literatura utilizando como base de dados o PubMed a fim de identificar os relatos e series de casos de embolias gordurosas em pacientes vítimas de traumas de natureza não ortopédica. A pesquisa final resultou em 23 artigos de pacientes que apresentaram embolia gordurosa/SEG devido a trauma não ortopédico.

**Resultados:**

a apresentação e a etiologia dessas embolias gordurosas são variadas e complexas, diferindo de paciente para paciente. Nesta revisão, destacamos a importância de manter uma suspeita clínica de SEG para pacientes vítimas de trauma que se encontrem sob cuidados intensivos.

**Conclusão:**

para ajudar os cirurgiões de trauma e os clínicos a identificar casos de SEG em pacientes com trauma que não apresentam fratura de ossos longos, foram destacados os principais sinais clínicos de SEG, bem como as possíveis opções de tratamento e prevenção.

## INTRODUCTION

Fat embolism was first described by Zenker in 1862 and later clinically diagnosed by Von Bergmann in 1873^1^. It is a complex phenomenon defined by the existence of fat particles in the microcirculation^2^ ^,^ ^3^. Fat embolism syndrome (FES), on the other hand, refers to the many possible clinical manifestations occurring due to fat embolism^4^. Fat embolism frequently occurs among trauma patients, more specifically, orthopedic trauma patients^2^ ^,^ ^4^ ^,^ ^5^. However, fat embolism has also been described in a variety of non-orthopedic-related trauma cases, such as burns, lung transplants, and liposuction^6^ ^,^ ^7^.

The presentation and diagnosis of FES are not yet well understood, and there are challenges in detecting the syndrome and precisely determining the related complications^6^. The diagnosis of fat embolism can be relatively difficult, as there are no all-inclusive criteria for its diagnosis; therefore, its diagnosis is usually one of exclusion^6^ ^,^ ^8^. Given the nature of circulating fat globules in the microcirculation, a diagnosis of fat embolism can be and is routinely performed during autopsy^6^ ^,^ ^9^. The underdiagnosis of fat embolism is highlighted by the fact that the clinical incidence was detected to be less than 1%, while postmortem incidence was evaluated to be 20%^10^ ^,^ ^11^. FES can involve multiple organs and is considered a lethal complication among trauma patients^12^. FES can lead to complications such as severe respiratory failure or brain death^13^. The mortality rate associated with FE and FES was estimated to range between 5% and 15%^14^ ^,^ ^15^. Therefore, due to the fatality of FES in trauma patients, it is important to maintain a high clinical suspicion of FES^16^.

Due to the relatively rare occurrence of FES in non-orthopedic trauma patients, it remains largely undiagnosed. We suggest that several factors, including the lack of standardized diagnostic criteria^14^ ^,^ ^17^, have played a key role in the underdiagnosis of fat embolism, especially in non-orthopedics-related trauma patients. Most cases of FES are associated with orthopedic patients with long bone fractures and pelvic fractures^2^ ^,^ ^12^ ^,^ ^14^ ^,^ ^17^ ^,^ ^18^. However, a few but significant number of cases are associated with non-orthopedic trauma^10^ ^,^ ^19^.

According to the findings in the literature, the pathophysiologic mechanism behind fat embolism in cases without any fractures might be attributed to two factors: first, the acute rise in pressure at the site of trauma, and second, the changes in the emulsification of blood lipids during shock^20^ ^,^ ^21^. FES is acknowledged as a fatal consequence of trauma, but with prompt identification and timely intervention, a positive prognosis is possible^12^. The aim of this paper is to highlight the importance of always suspecting fat embolism in trauma cases, including those that are not exclusively orthopedic cases. We will discuss the challenges of diagnosing this complication and the ways to treat it and reduce the mortality related to FES.

## METHODS

A literature search was conducted using the PubMed database. The PubMed search was conducted using the terms fat embolism and fat embolism syndrome (MeSH). This initial search produced 4175 papers. We narrowed the search to case reports published from September 1^st^, 2013, to September 1^st^, 2023. This yielded 289 case reports.

We further investigated these papers and excluded all papers of patients presenting with a fat embolism or FES due to orthopedic-related trauma. When we categorized the related medical conditions, we defined orthopedic trauma as fractures and interventions such as total hip replacement, knee arthroplasty, internal fixation, and spinal instrumentation. Additionally, any bone fracture due to blunt force trauma, falls, and motor collisions was classified as orthopedic trauma. On the other hand, non-orthopedic trauma encompassed procedures such as liposuction or fat injection, as well as medical conditions such as burns, bone marrow harvesting and transplant, and soft tissue injuries, as indicated in Table 1. The final number of case reports relevant to the study was found to be 21.

**Table 1:** Non-orthopedic diagnosis and procedures with potential for fat embolism.

Non-Orthopedic Trauma
Liposuction
Soft Tissue Injury
Severe Burns
Crush Injuries
Bone marrow harvesting and transplant

Additionally, papers of patients from other search engines, such as Google Scholar, were found and added to the total number of relevant papers. This brought the total number of relevant papers of patients presenting with a fat embolism or FES due to non-orthopedic-related trauma to be 23 papers.

For the clinical presentation, we referred to respiratory abnormalities as the presence of tachypnea, hypoxemia, dyspnea, chest pain, hemoptysis, and/or cyanosis. Neurological abnormalities were characterized by the presence of disturbance of consciousness, restlessness, seizures, limb weakness, paralysis, aphasia, sensory disturbance, headache, and/or dizziness, Cardiovascular abnormalities were identified by the presence of tachycardia, hypotension, cardiac arrest, bradycardia, and/or angina, as presented in Table 2.

**Table 2:** Main clinical signs and symptoms related to FES.

Organ/System compromised	Main symptoms
Respiratory	Tachypnoea Hypoxaemia ARDS (acute respiratory distress syndrome)
Neurological	Confusion Seizures Altered level of consciousness Focal neurological deficits
Dermatological	Petechial rash
Systemic	Fever
Cardiovascular	Tachycardia Hypotension Arrhythmia Myocardial ischaemia Pulmonary hypertension Right-sided heart failure
Ophthalmic	Purtscher’s retinopathy (cotton wool exudates, macular oedema and haemorrhage)
Organ/System compromised	Main symptoms
Renal	Oliguria Proteinuria Lipiduria Haematuria
Hepatic	Jaundice
Haematological	Anaemia Thrombocytopenia Coagulopathy Fat macroglobulinaemia

## RESULTS

In our search, several case reports were identified to have fat embolism or FES in patients experiencing non-orthopedic-related trauma. We identified 23 papers discussing case presentations of fat embolism from patients presenting with conditions such as soft tissue injury with no fracture and liposuction. The main findings related to the cases in this review are presented in Table 3.

**Table 3:** Summary of case report findings with their respective clinical signs, diagnostic criteria, treatment, and outcomes.

Case	Underlying Cause	Main Clinical Presentation	Main Radiological Findings	Diagnostic Tool	Treatment	Outcome
Xu L et al. 2023^22^	Soft tissue injury due to silver-needle acupuncture therapy	-Dizziness/ fatigue -Anormal mental status -Significant Hypotension	-ECG: ST-T changes -CT: widening of inf pulmonary artery and RA, LV, RV	Autopsy	N/A	Death
Pham 2022^23^	Plastic surgery (breast surgery and abdominal liposuction)	-mild dyspnea -decreased O2 saturation -chest tightness/fatigue	- CT: fat embolus in right pulmonary artery	Chest CT	Anticoagulants	Patient discharged postoperative day 7
Wolfe et al. 2022^24^	Case 1: BBL surgery (Brazilian Butt Lift)	Case 1: -Hypotension -Tachycardia -Cyanosis -Hypoxia -Severe respiratory acidosis	Case 1: -CT: multiple pulmonary emboli with diffuse ground-glass opacities	Case 1: Chest CT	Case 1: Norepinephrine (NE) infusion and 100% fraction of 02. Required 6 days of ECMO.	Patient discharged Postoperative day 24 with tunneled hemodialysis catheter and was listed for renal transplantation due to kidney failure.
	Case 2: Liposuction with fat transfer	Case 2: -Respiratory distress -Hypoxia -Severe respiratory acidosis	CCase 2: -CT: pulmonary emboli and splenic and renal micro emboli -Brain CT: hypodensity of right middle cerebral artery	Case 2: Chest CT	Case 2: Intubation	Patient discharged after 10 days of physical therapy. Patient returned due to post-operative complications after 1 month.
Case	Underlying Cause	Main Clinical Presentation	Main Radiological Findings	Diagnostic Tool	Treatment	Outcome
Kadar et al. 2021^25^	Elective Liposuction	-Respiratory failure -Altered mental status -Tachycardia -Low O2 saturation	-ECG: right axis deviation and ST-T changes -CT: bilateral diffuse ground-glass lung opacities -Brain CT: diffuse edema and infarction	Bronchoa veolar lavage (BAL)	Positive airway pressure, antibiotics, intubation, and mechanical ventilation	Patient transferred from ICU to general medicine unit on hospital day 7 with moderate cognitive impairment which improved after 1 month follow-up.
Dong et al. 2021^26^	Trauma craniotomy after breast and hip augmentation	-disturbance of consciousness -convulsion of right upper extremity -intracranial hypertension	no chest CT or echocardiogram abnormalities	Fat embolus found during trauma craniotomy	Transfer to neuro ICU after surgery.	Patient discharged on hospital day 30 with regained cognitive function.
Dhooghe et al. 2022^27^	Facial fat graft	N/A	N/A	Autopsy	N/A	Death
Uz et al. 2020^28^	Buttock Hyaluronic acid filler injection	-agitation/altered mental status -low O2 saturation -decreased respiratory sounds	Chest CT: bilateral ground-glass opacities and pleural effusion	Brain CT: multiple millimetric hypertense foci	Enoxaparin and methylprednisolone	Discharged after 20 days in the hospital
Wang et al. 2020^29^	Liposuction and fat injection	-hypoxemia -tachycardia -respiratory acidosis	Chest CT: multiple filling defects bilaterally and patchy shadows	CT findings	Anticoagulants and steroids, oxygen therapy	Patient discharged after 18 days in the hospital.
Meng et al. 2020^30^	Hit by a car (no fracture)	-unconsciousness	N/A	Autopsy	N/A	Death
Lee et al. 2020^31^	Autologous fat grafting	-hemiparesis -hemisensory deficits	MRA: acute occlusion of right middle cerebral artery	Microscopic findings of aspirated material	Aspirational thrombectomy	Patient discharged after 5 days in hospital. Recovered neurological deficits after 1 year.
Case	Underlying Cause	Main Clinical Presentation	Main Radiological Findings	Diagnostic Tool	Treatment	Outcome
Lu 2019^32^	Autologous fat graft	-seizures and collapse	Ultrasonography: solid echogenic material in carotid	Digital subtraction angiography revealed fat particles.	N/A	Patient died after 20 days of diagnosis due to massive bilateral cerebral infarction
Peña et al. 2019^33^	Liposuction and fat injection	-low O2 saturation -hypotension	-Echo: RV dysfunction -Chest CT: ground glass opacities	Chest CT	Intubation and tracheostomy	Patient stayed in ICU for 80 days with Acute Respiratory Distress Syndrome (ARDS). At discharge, neurological function was normal but intensive
Rosenfeld et al. 2019^34^	2 cases: Transplantation using marginal steatotic liver grafts.	-low oxygenation postsurgery - Pulseless Electrical Activity (PEA)	-Chest X ray: diffuse airspace/perihilar opacities	Autopsy	N/A	Death
Zhibin et al. 2018^35^	Liposuction	-Deterioration of consciousness -Left hemialgia -Petechiae after 14 hr	-MRI: large areas of hyperintense foci -Brain CT: low intensity signal in left hemisphere -Chest CT: normal	Clinical presentation and CT/MRI findings	N/A	Patient died after her condition deteriorated due to hepatic failure, acute renal insufficiency, pneumonia, and cerebral herniation.
Scarpino et al. 2018^36^	Off-pump CABG	-chest pain -Dyspnea	Brain CT: presence of hypodense lesions with the range of fat	MRI: hyperintense lesions in same areas as CT	N/A	Patient transferred to ICU with major neurological deficits. No further information was given.
Ali et al. 2017^37^	Liposuction	Triad of: -respiratory failure -cerebral dysfunction -petechial rash	Chest X-ray: bilateral generalized nfiltrates	Clinical presentation and history	Atropine, intubation, and organ support	Patient discharged 14 days after extubation with good recovery.
Cantu and Pavlisko 2017^38^	Liposuction	-Dyspnea -Amaurosis -Hypoxemia	Chest CT was contraindicated	Autopsy	N/A	Death
Case	Underlying Cause	Main Clinical Presentation	Main Radiological Findings	Diagnostic Tool	Treatment	Outcome
Sato et al. 2016^39^	Liposuction and abdominoplasty	-Fever -Dyspnea -Bilateral papilledema	MRI: restriction in diffusion	Surgical history	Anticoagulant	Amaurosis was not reversed with anticoagulants.
Mendoza-Morales et al. 2016^40^	Injection of Vitamin E	-Respiratory distress -Acute Renal Failure -Neurological impairment	Tomography: cerebraledema	Autopsy	N/A	Death
Jacob et al. 2016^41^	2 cases due Donor-acquired FE after lung transplantation	Case 1: Hypoxemic respiratory failure	Case 1: Chest CT: consolidation in donor lung	Case1: Histological evaluation of the donor lung	Case 1: Ventilation and tracheostomy	Discharged after prolonged respiratory failure to a acute rehabilitation facility.
		Case 2: Severe hypoxemia and frothy secretions	Case 2: Chest CT: patchy opacities	Case2: CT and intraoperative findings	Case 2: ECMO	Discharged after prolonged respiratory failure to an acute rehabilitation facility. However, patient died due to transplant rejection.
Berger et al. 2013^42^	Gunshot Wounds with no fracture	N/A	N/A	Autopsy	N/A	Death
Bajraktari et al. 2022^43^	Minimal Soft Tissue Trauma Due to a Motor Vehicle Collision with no fractures	-Hypoxemia -Petechia -Conjunctival pallor -Confusion	-Chest X-ray : diffuse infiltrates -Chest CT: significant consolidations	Bronchoalveolar lavage (BAL)	-Mechanical ventilation -Supportive care	Patient discharged after 15 days in the hospital.
Fowler et al. 2021^44^	Motor Vehicle accident with no fractures	-Left facial droop -Left upper extremity drift	-Brain CT: Hyperdense MCA	Pathological analysis of suctioned thrombus	Monitoring in ICU	Patient discharged with outpatient physical therapy.

Among all the cases we found, there was a diverse range of clinical presentations, risk factors, and ages of incidence. In our search, the most common cause of non-orthopedic trauma-related patients was cosmetic procedures such as liposuction. We present our findings in Table 3.

Due to the inconsistencies among current diagnostic criteria, difficulties arise in the clinical setting for the diagnosis of FES. Summaries of the common diagnostic criteria used for FES are displayed in Tables 4, 5, and 6.

**Table 4:** Key Distinct Components for Diagnosing a Fat Embolism based on the Gurd criteria.

Key Clinical Manifestation	Gurd Classification
Petechial Rash	Major Criteria
Respiratory Insufficiency and	Major Criteria
Cerebral Involvement not related to head trauma	Major Criteria
Fever >38.5	Minor Criteria
Tachycardia >110bpm	Minor Criteria
Retinal Involvement	Minor Criteria
Jaundice	Minor Criteria
Renal Signs	Minor Criteria
Anemia	Minor Criteria
Thrombocytopenia	Minor Criteria
High Erythrocyte Sedimentation Rate	Minor Criteria
Fat Macroglobulinemia	Minor Criteria

Diagnosis requires 2 major criteria OR at least 1 major criterion and 4 minor criteria

**Table 5:** Schonfeld Classification System for Fat Embolism Syndrome

Clinical Manifestation	Number of Points
Petechial Rash	5
Diffuse Infiltrate on X-ray	4
Hypoxemia	3
Fever, tachycardia, confusion	1 (each)

A diagnosis of FES is made when a cumulative score greater than 5 is found^45^

**Table 6:** Summary of Linedgue Diagnostic Criteria for Fat Embolism Syndrome

Clinical Manifestations	Criteria for Diagnosis
Sustained pO2 <60mmHg	The presence of at least one of these clinical manifestations leads to a diagnosis of FES
Sustained pCO2 >55mmHg OR a pH of less than 7.3	
Sustained Respiratory rate >35 per min even after given sedation	
Increased work of breathing, tachycardia, and anxiety	

An additional tool to aid in the diagnosis of FES is the use of imaging. Common radiological imaging tools and their expected clinical picture within FES are summarized in Table 7.

**Table 7:** Summary of Common Radiological Imaging Findings in Fat Embolism Syndrome.

Imaging Tool	Expected Findings
Chest X-ray	Diffuse Interstitial Infiltrates^2^
	Pulmonary edema^3^
Chest CT Scan	Diffuse Areas with Pulmonary Edema and Vascular Congestion^2^
	Patchy ground-glass opacities^46^
	Small Bilateral Pleural Effusions^46^
Cerebral MRI (For Cerebral Fat Embolism)	Bright Spots on a Dark Background (Starfield Pattern) and microbleeding^36^

The large variance among these diagnostic criteria, as seen in Tables 3-5, may lead to irregularity in the diagnosis of FES. A patient fulfilling one of the criteria (e.g., Gurd) may not fulfill one of the other criteria (e.g., Schonfeld) despite presenting with a fat embolism.

## DISCUSSION

### Pathophysiology

Several theories exist that attempt to explain the full pathophysiology of FES; however, they inadequately encompass all presentations associated with FES^47^. The current pathophysiological understanding of fat embolism is dominated by two differing theories: biochemical theory and mechanical theory. The biochemical theory is built on the understanding that clinical manifestations of fat embolism occur due to a proinflammatory environment^4^. The biochemical theory speculates that trauma causes an inflammatory response in the body that leads to the release of fat cells from the marrow of the bone into the venous system^2^. Neutral fat found in the bone marrow does not normally cause acute lung injury; however, it could be metabolized over several hours into intermediates such as free fatty acids that could cause damage and lead to clinically relevant sequelae such as acute respiratory distress syndrome (ARDS), as was seen in animal models^48^. Free fatty acids could also potentially lead to complications such as dysfunction of cardiac muscle^48^. The biochemical theory could potentially explain delays in the clinical manifestations of FES^9^. The other predominant theory is the mechanical theory. The mechanical theory states that an increase in intramedullary pressure due to trauma can cause the release of fat into the venous system via open venous sinusoids^2^. The biochemical theory assists in explaining FES for nontraumatic reasons^4^. The FES seen in most patients is most likely due to a combination of both biochemical and mechanical theories^4^.

To understand the basis of FES conception, a further scrutiny of the biochemical and mechanical theories is needed to correlate what occurs at the cellular level and what is observed clinically. The biochemical theory (or the Sedimentation theory) puts forward that an increase in catecholamine levels and plasma lipase allows lipids from fat stores in the body to mobilize, eventually forming fat droplets within the circulation^44^ ^,^ ^49^. The biochemical theory may be summarized in the following manner: any form of trauma to the body triggers a pro-inflammatory response which aims to advance cellular repair and to promote readiness for potential further traumatic insults. Following this, the body may sometimes form an exaggerated response to subsequent ensuing trauma such as a fat embolism. This amplified response is theorized to add additional endothelial cellular injury leading to possible multi organ damage as seen in FES^7^.

The mechanical theory postulates that fat stored in the bone marrow obtains access to the circulation via venous sinusoids in the presence of trauma^4^. These fat particles then travel into the vasculature and embolize causing the symptoms of FES^44^. The mechanical theory does not explain the full series of manifestations seen in FES^44^. It fails to explain the temporal separation of events seen typically 24-72 hours after a fat embolism such as a petechial rash^7^.

In addition to the biochemical theory and the mechanical theory, the coagulation theory has also been proposed to elucidate the pathophysiology of FES. The coagulation theory states that tissue thromboplastin that is released by the marrow activates both the complement system and the extrinsic coagulation cascade^10^. This is done by direct activation of Factor VII which causes intravascular coagulation to occur^10^. The coagulation theory proposes that the circulating fat causes an inflammatory environment^7^. The presence of hypovolemia that is typically seen after trauma, endothelial damage, and the aforementioned inflammatory environment lead to the activation of the clotting cascade, which may potentially increase the size of the fat embolism thereby intensifying obstruction in the circulation^7^. 

In conclusion, the three proposed theories for fat embolism explain, by different means, the possible sequence of events leading to the clinical manifestations seen in FES. It becomes clear that no singular theory may account for all the manifestations seen in FES, and that it is more likely an amalgamation of all three theories that work synergistically resulting in the clinical phenomena seen in FES.

### Incidence

The exact incidence of fat embolism remains unknown^50^. It varies significantly in the literature depending on the cause and the diagnostic criteria used. The incidence was reported to be as low as less than 1% in some studies^51^ and as high as 20% in others^11^ ^,^ ^52^. Most of the studies explore the incidence of FES in orthopedic patients and not in patients presenting with FES due to non-orthopedic-related trauma. He et al. reported in their pooled analysis from PubMed and Web Science the incidence of FES in nonfracture trauma-related cases^19^. A total of 11.8% of FES cases were associated with liposuction or autologous fat injection, 2.2% of cases were associated with fat-soluble injections, and 0.7% were associated with multiple soft tissue injuries^19^. Non-orthopedic fat embolism has been associated with cosmetic procedures such liposuctions and fat grafting, and it happens most commonly in the lungs. It can be explained by the generation of lipid fragments that enter the venous circulation following damage to adipose tissue and small blood vessels, which results in lung injury^53^. The survival rate for patients with multiple soft tissue injuries was reported to be zero in this paper^19^. While the incidence of FES is significantly low in cases of multiple soft tissue injuries, the fatality is very high, which is why it is important for physicians to be alert about the presentation of FES.

### Imaging and Clinical Investigations

Adjunct to clinical assessment, investigations and diagnostic tests can support the diagnosis of FES. Arterial blood analysis (ABG) with Pa02 of less than 60mmHg and hypocapnia demonstrating an increase in pulmonary shunt fraction and an A-a gradient increase strongly suggest FES^50^. Additionally, a decrease in hematocrit 24 to 48 hours post trauma is also suggestive of FES, as it can be due to intra-alveolar hemorrhage^50^. Cytological examination of urine showing fat globules is not specific^50^. However, a study showed that cytological examination of pulmonary capillary blood from a wedged pulmonary artery catheter can be useful for the early detection of FES^54^.

Imaging can also be helpful in confirming FES diagnosis. Chest CT with ground-glass opacities is the most common finding in FES patients^19^. A chest X-ray with patchy infiltrative shadow or low transmittance can also suggest FES^19^. However, a normal initial chest CT or chest X ray does not rule out FES diagnosis, and it is important to keep in mind the risks associated with repeated radiological exposure when considering a second CT scan^16^. A more telling imaging test is a cerebral MRI revealing dispersed hyperintense lesions on T2 images^16^. It has been demonstrated to be sensitive in FES patients with cerebral manifestations, including those with normal chest CT results^10^ ^,^ ^55^. It has also been reported in the literature that cerebral fat embolism presents with microbleeds exhibiting a characteristic patten described as “walnut kernel”, which can be helpful in diagnosing difficult cases of FES^56^. The use of bronchoalveolar lavage (BAL) to support FES diagnosis remains controversial. Although the detection of fat droplets in alveolar macrophages can enable early and rapid diagnosis of FES^57^, it is not specific, as fat droplets in alveolar macrophages can also be associated with lipid infusions, sepsis, or hyperlipidemia^50^.

### Diagnostic Criteria

Diagnosing FES is still very challenging and relies mainly on a combination of clinical symptoms and imaging and laboratory findings^58^ ^,^ ^59^. There are multiple diagnosis criteria for FES: Gurd and Wilson criteria, Modified Gurd criteria, Schonfeld criteria, and Lindeque criteria, summarized in Tables 3-5^16^. Most of these criteria have been criticized in the literature due to their low specificity^55^. They are all rooted in the classical triad of progressive respiratory insufficiency, petechial rash, and mental deterioration that manifest 24-48 hours post trauma^16^. However, studies report that the presentation of the three triad criteria simultaneously is very low^16^ ^,^ ^55^ ^,^ ^60^. He et al. reported that the two most common clinical symptoms are respiratory abnormalities (34.6%), which present as hypoxemia, dyspnea, and tachypnea, and neurological disturbances (27.3%), which manifest as disturbance of consciousness^19^. Cutaneous manifestations, such as petechial rash, are less commonly observed^19^. Additionally, studies have shown that the petechial rash does not appear until 3-5 days after the onset of respiratory insufficiency^10^ ^,^ ^46^. Therefore, the combination of respiratory insufficiency and neurological disturbances can be sufficient to suspect FES and diagnose it, which is indicated by the Gurd and Wilson criteria as well as the modified Gurd criteria^16^. Aggarwal et al. recommend suspecting FES and referring patients to the ICU when they present with neurological disturbances and hypoxemia along with long bone fractures^12^. Future areas of research focusing on FES should involve enhancing our comprehension of FES to allow for a more accurate diagnosis^58^. 

### Treatment and Management

The treatment and management course for fat embolism has not been well established and remains chiefly supportive in nature. Therapeutic interventions made specifically for tackling FES have mostly been ineffective^2^. Corticosteroids, which work via several mechanisms, such as potentially decreasing the levels of free fatty acids, may aid in the treatment of FES^2^. There has been some evidence that corticosteroids may be effective in the prevention of FES in patients with long bone fractures^61^. However, the use of corticosteroids in the management and treatment of fat embolism remains a controversial topic. Additional proposed pharmacological interventions include the use of systemic anticoagulation therapy for patients with FES^4^. However, the use of heparin to treat FES carries serious potential complications, such as bleeding, that must be taken into account and could possibly be a dangerous addition to the treatment regimen of FES^47^ ^,^ ^62^. Another potential preventative treatment for fat embolism is the use of early internal fixation devices for patients with long bone fractures, which could potentially reduce the incidence of FES^3^. The delayed stabilization of fractures has been associated with an increased risk of pulmonary insults such as fat embolism^63^.

Supportive care continues to be the key pillar in the management of FES. A key aspect to the management of fat embolism is ensuring sufficient arterial oxygenation^50^. Patients need to be provided oxygen to maintain the PaO_2_ of oxygen at 90mmHg or higher^64^. If the hypoxemia is severe and approaches dangerous levels, endotracheal intubation and additional mechanical ventilation support are given^64^. Several case studies have been presented with patients presenting with FES due to non-orthopedic trauma-related reasons who have successfully been treated due to a combination of therapeutic strategies that include mechanical and pharmacological interventions. One case report of a 29-year-old woman presenting with FES while undergoing liposuction was successfully treated with no long-term complications due to a combination of several therapeutic interventions, including ventilation, a low dose course of corticosteroids, human albumin, and low-weight molecular heparin^65^. The use of venovenous extracorporeal membrane oxygenation (VV-ECMO) was recently proven to be successful in managing patients with acute respiratory distress caused by FES^66^ ^,^ ^67^. However, data and information encompassing the use of these therapeutic techniques in non-orthopedic trauma-related FES cases are sparse and demand more attention to fully understand effective treatments.

### Limitations

The study’s limitations include a few factors. Firstly, the prevalence and small sample size of the searched papers may affect the generalizability of the findings. Since the study is based on a limited number of papers, it may not capture the full spectrum of the condition or accurately represent the population at large. Additionally, due to the lack of specific treatment options, the study may not provide clear guidance on managing the condition effectively. Moreover, the reliance on case reports in the published literature suggests that there is a significant number of misdiagnosis patients, which may further influence the validity and reliability of the findings. It is important to take these limitations into consideration when interpreting the results and applying them to clinical practice.

## CONCLUSION

FES remains undiagnosed in many trauma cases and the related fatality is very high. In many cases, FES is only discovered as the cause of death on postmortem autopsy. Treatment remains limited and continues to be mainly supportive, and interventions such as maintaining sufficient oxygen levels are crucial for patient survival. Therefore, it is important to be clinically alert and suspect of FES when a trauma patient presents with respiratory insufficiency and neurological disturbances.

## References

[1] E Von Bergmann (1873). Ein fall todlicher fettembolie.

[2] Kwiatt ME, Seamon MJ (2013). Fat embolism syndrome. Int J Crit Illn Inj Sci.

[3] Adeyinka A, Pierre L (2023). StatPearls.

[4] Kosova E, Bergmark B, Piazza G (2015). Fat embolism syndrome. Circulation.

[5] Lempert M, Halvachizadeh S, Ellanti P, Pfeifer R, Hax J, Jensen KO (2021). Incidence of Fat Embolism Syndrome in Femur Fractures and Its Associated Risk Factors over Time-A Systematic Review. J Clin Med.

[6] Timon C, Keady C, Murphy CG (2021). Fat Embolism Syndrome - A Qualitative Review of its Incidence, Presentation, Pathogenesis and Management. Malays Orthop J.

[7] Luff D, Hewson DW (2021). Fat embolism syndrome. BJA Educ.

[8] Shaikh N, Parchani A, Bhat V, Kattren MA (2008). Fat embolism syndrome clinical and imaging considerations: case report and review of literature. Indian J Crit Care Med.

[9] Milroy CM, Parai JL (2019). Fat Embolism, Fat Embolism Syndrome and the Autopsy. Acad Forensic Pathol.

[10] George J, George R, Dixit R, Gupta RC, Gupta N (2013). Fat embolism syndrome. Lung India.

[11] Georgopoulos D, Bouros D (2003). Fat embolism syndrome clinical examination is still the preferable diagnostic method. Chest.

[12] Aggarwal R, Banerjee A, Soni KD, Kumar A, Trikha A (2019). Clinical characteristics and management of patients with fat embolism syndrome in level I Apex Trauma Centre. Chin J Traumatol.

[13] Aggarwal R, Pal S, Soni KD, Gamangatti S (2015). Massive cerebral fat embolism leading to brain death A rare presentation. Indian J Crit Care Med.

[14] Kainoh T, Iriyama H, Komori A, Saitoh D, Naito T, Abe T (2021). Risk Factors of Fat Embolism Syndrome After Trauma A Nested Case-Control Study With the Use of a Nationwide Trauma Registry in Japan. Chest.

[15] Fulde GW, Harrison P (1991). Fat embolism--a review. Arch Emerg Med.

[16] Nixon MJ, Grant T (2021). Subacute fat embolism syndrome in a young female trauma patient during COVID-19. J Surg Case Rep.

[17] Stein PD, Yaekoub AY, Matta F, Kleerekoper M (2008). Fat embolism syndrome. Am J Med Sci.

[18] White T, Petrisor BA, Bhandari M (2006). Prevention of fat embolism syndrome. Injury.

[19] He Z, Shi Z, Li C, Ni L, Sun Y, Arioli F, Wang Y, Ammirati E (2021). Single-case metanalysis of fat embolism syndrome. Int J Cardiol.

[20] Li S, Zou D, Qin Z, Liu N, Zhang J, Li Z (2015). Nonfracture-associated pulmonary fat embolism after blunt force fatality case report and review of the literature. Am J Forensic Med Pathol.

[21] Nichols GR, Corey TS, Davis GJ (1990). Nonfracture-associated fatal fat embolism in a case of child abuse. J Forensic Sci.

[22] Xu L, Tan X, Chen X, Du S, Yue X, Qiao D (2023). Rare, fatal pulmonary fat embolism after acupuncture therapy A case report and literature review. Forensic Sci Int.

[23] Pham MQ (2022). Fat Embolism After Plastic Surgery A Case Report. Plast Aesthetic Nurs.

[24] Wolfe EM, Weber LE, Wo LM, Samaha MJ, Mathew P, Garcia O (2022). Two Cases Surviving Macro Fat Emboli Complications Following Gluteal Fat Grafting. Aesthet Surg J.

[25] Kadar A, Shah VS, Mendoza DP, Lai PS, Aghajan Y, Piazza G (2021). Case 39-2021: A 26-Year-Old Woman with Respiratory Failure and Altered Mental Status. N Engl J Med.

[26] Dong W, Wan D-Y, Yang X, Fu M, Liu X, Li H (2021). Delayed onset of fat embolus in the cerebral venous system after breast and hip augmentation a case report. BMC Neurol.

[27] Dhooghe NS, Maes S, Depypere B, Claes KEY, Coopman R, Kubat B (2022). Fat Embolism After Autologous Facial Fat Grafting. Aesthet Surg J.

[28] Uz I, Yalçinli S, Efe M (2020). Fat embolism syndrome after gluteal augmentation with hyaluronic acid A case report. Ulus Travma Acil Cerrahi Derg.

[29] Wang C, Wang X, Huang J, Yu N, Long X (2020). Severe fat embolism after autologous fat grafting in vaginal tightening and breast augmentation surgery. J Int Med Res.

[30] Meng Y, Zhang M, Ling H, Huang S, Miao Q, Yu Y (2020). Nontraumatic Multiple-Organ Fat Embolism An Autopsy Case and Review of Literature. Am J Forensic Med Pathol.

[31] Lee HS, Park J-J, Roh HG, Lim SD (2020). Unusual clinicopathological presentation of nontraumatic cerebral fat embolism Two-case report. Medicine (Baltimore).

[32] Lu Y-Q (2019). Young Woman With Seizures. Ann Emerg Med.

[33] Peña W, Cárdenas-Camarena L, Bayter-Marin JE, McCormick M, Durán H, Ramos-Gallardo G (2019). Macro Fat Embolism After Gluteal Augmentation With Fat First Survival Case Report. Aesthet Surg J.

[34] Rosenfeld DM, Smith ML, Seamans DP, Giorgakis E, Gaitan BD, Khurmi N (2019). Fatal diffuse pulmonary fat microemboli following reperfusion in liver transplantation with the use of marginal steatotic allografts. Am J Transplant.

[35] Zhibin Z, Peng S, Fang C (2018). Fat embolism following a liposuction procedure. Neurol India.

[36] Scarpino M, Lanzo G, Moretti M, Olivo G, Amantini A, Grippo A (2018). Delayed cerebral fat embolism occurring after off-pump coronary artery bypass grafting. Cardiol J.

[37] Ali A, Theobald G, Arshad MA (2017). Fat attacks a case of fat embolisation syndrome postliposuction. BMJ Case Rep.

[38] Cantu CA, Pavlisko EN (2017). Liposuction-induced fat embolism syndrome. BMJ Case Rep.

[39] Sato HK, Kowacs PA, Dalmau J, Santos PSF (2016). Fat embolism showing restriction on diffusion sequence in brain magnetic resonance imaging. Arq Neuropsiquiatr.

[40] Mendoza-Morales RC, Camberos-Nava EV, Luna-Rosas A, Garcés-Ramírez L, De la Cruz F, García-Dolores F (2016). A fatal case of systemic fat embolism resulting from gluteal injections of vitamin e for cosmetic enhancement. Forensic Sci Int.

[41] Jacob S, Courtwright A, El-Chemaly S, Racila E, Divo M, Burkett P (2016). Donor-acquired fat embolism syndrome after lung transplantation. Eur J Cardio-Thorac Surg.

[42] Berger N, Paula P, Gascho D, Flach PM, Thali MJ, Ross SG (2013). Bone marrow edema induced by a bullet after a self-inflicted accidental firing. Leg Med (Tokyo).

[43] Bajraktari M, Naco M, Huti G, Arapi B, Domi R (2022). Fat Embolism Syndrome Without Bone Fracture Is It Possible?. Open Access Maced J Med Sci.

[44] Fowler JB, Fiani B, Sarhadi K, Cortez V (2021). Cerebral fat embolism in the absence of a long bone fracture A rare case report. Surg Neurol Int.

[45] Schonfeld SA, Ploysongsang Y, DiLisio R, Crissman JD, Miller E, Hammerschmidt DE (1983). Fat embolism prophylaxis with corticosteroids A prospective study in high-risk patients. Ann Intern Med.

[46] Newbigin K, Souza CA, Torres C, Marchiori E, Gupta A, Inacio J (2016). Fat embolism syndrome State-of-the-art review focused on pulmonary imaging findings. Respir Med.

[47] Mellor A, Soni N (2001). Fat embolism. Anaesthesia.

[48] Gupta A, Reilly CS (2007). Fat embolism. Contin Educ Anaesth Crit Care Pain.

[49] Bardana D, Rudan J, Cervenko F, Smith R (1998). Fat embolism syndrome in a patient demonstrating only neurologic symptoms. Can J Surg.

[50] Shaikh N (2009). Emergency management of fat embolism syndrome. J Emerg Trauma Shock.

[51] Bulger EM, Smith DG, Maier RV, Jurkovich GJ (1997). Fat embolism syndrome A 10-year review. Arch Surg.

[52] Fabian TC, Hoots AV, Stanford DS, Patterson CR, Mangiante EC (1990). Fat embolism syndrome prospective evaluation in 92 fracture patients. Crit Care Med.

[53] Kao Y-M, Chen K-T, Lee K-C, Hsu C-C, Chien Y-C (2023). Pulmonary Fat Embolism Following Liposuction and Fat Grafting A Review of Published Cases. Healthc Basel Switz.

[54] Van den Brande FGJ, Hellemans S, De Schepper A, De Paep R, Op De Beeck B, De Raeve HR, Jorens PG (2006). Post-traumatic severe fat embolism syndrome with uncommon CT findings. Anaesth Intensive Care.

[55] Godoy DA, Di Napoli M, Rabinstein AA (2018). Cerebral Fat Embolism Recognition, Complications, and Prognosis. Neurocrit Care.

[56] Giyab O, Balogh B, Bogner P, Gergely O, Tóth A (2021). Microbleeds show a characteristic distribution in cerebral fat embolism. Insights Imaging.

[57] Vedrinne JM, Guillaume C, Gagnieu MC, Gratadour P, Fleuret C, Motin J (1992). Bronchoalveolar lavage in trauma patients for diagnosis of fat embolism syndrome. Chest.

[58] Rothberg DL, Makarewich CA (2019). Fat Embolism and Fat Embolism Syndrome. J Am Acad Orthop Surg.

[59] Kawakami D, Yoshino S, Kawakami S, Yamakawa R (2022). Fat embolism syndrome. Intensive Care Med.

[60] Alfudhili K (2018). Pearls in Pulmonary Computed Tomography Findings in Patients With Fat Embolism Syndrome. Can Assoc Radiol J.

[61] Bederman SS, Bhandari M, McKee MD, Schemitsch EH (2009). Do corticosteroids reduce the risk of fat embolism syndrome in patients with long-bone fractures A meta-analysis. Can J Surg.

[62] Piastra M, Picconi E, Morena TC, Ferrari V, Gelormini C, Caricato A (2023). Multisystemic involvement of post-traumatic fat embolism at a Pediatric Trauma Center a clinical series and literature review. Eur J Pediatr.

[63] Bone LB, Johnson KD, Weigelt J, Scheinberg R (1989). Early versus delayed stabilization of femoral fractures A prospective randomized study. J Bone Joint Surg Am.

[64] Richards RR (1997). Fat embolism syndrome. Can J Surg.

[65] Ding YJ, Zhang L, Sun XW, Lin YN, Li QY (2022). Rapid recovery of fat embolism syndrome with acute respiratory failure due to liposuction. Respirol Case Rep.

[66] Momii K, Shono Y, Osaki K, Nakanishi Y, Iyonaga T, Nishihara M (2021). Use of venovenous extracorporeal membrane oxygenation for perioperative management of acute respiratory distress syndrome caused by fat embolism syndrome A case report and literature review. Medicine (Baltimore).

[67] Lari A, Abdulshakoor A, Zogheib E, Assaf N, Mojallal A, Lari A-R (2020). How to Save a Life From Macroscopic Fat Embolism A Narrative Review of Treatment Options. Aesthet Surg J.

